# HBV and HDV: New Treatments on the Horizon

**DOI:** 10.3390/jcm10184054

**Published:** 2021-09-08

**Authors:** Valentina Zuccaro, Erika Asperges, Marta Colaneri, Lea Nadia Marvulli, Raffaele Bruno

**Affiliations:** 1 U.O.C. Malattie Infettive I Fondazione IRCCS Policlinico San Matteo–Università di Pavia, 27100 Pavia, Italy; e.asperges@smatteo.pv.it (E.A.); marta.colaneri01@universitadipavia.it (M.C.); leanadia.marvulli01@universitadipavia.it (L.N.M.); raffaele.bruno@unipv.it (R.B.); 2 Dipartimento di Scienze Clinico-Chirurgiche, Diagnostiche e Pediatriche–Università di Pavia, 27100 Pavia, Italy

**Keywords:** HBV, HDV, antivirals, functional cure, pharmacology

## Abstract

Despite the accumulating knowledge, chronic hepatitis B (CHB) and HDV infection represent a global health problem, and there are still several critical issues, which frequently remain uncovered. In this paper, we provided an overview of the current therapeutic options and summarized the investigational therapies in the pipeline. Furthermore, we discussed some critical issues such as a “functional cure” approach, the futility of long-term NA therapy and the relevance of understanding drug actions and safety of antivirals, especially in special populations.

## 1. Introduction

Viral hepatitis has been recognized as a health and development priority only recently [1]. Most countries have implemented neonatal vaccination programs against hepatitis B virus (HBV) and reached a reduction in HBV prevalence among children; despite this, the burden this infection places, especially on the adult population, is still huge. In 2015, an estimated 257 million people were living with chronic HBV infection (CHB) worldwide, and its complications (especially long-term consequences, i.e., cirrhosis and hepatocellular carcinoma) were responsible for the 66% of the deaths caused by viral hepatitis; future perspectives are worrying, with 17 million deaths attributable to CHB in 2030 [2,3]. It is estimated that 5% of HBV-infected persons are also coinfected with hepatitis Delta virus (HDV) and have a more severe liver disease; however, there is substantial uncertainty, as in many countries, HDV infection is not tested [2].

Currently, the recommended treatment of choice for CHB regardless of the severity of the liver disease is the long-term administration of a nucleos(t)ide analog (NA) with a high barrier to resistance, such as entecavir, tenofovir disoproxil fumarate and telbivudine; alternatively, for patients with mild to moderate CHB, a 48 week-therapy with peginterferon alfa (PegIFNa) can be considered [4]. The main endpoint of all current treatment strategies is suppression of HBV DNA levels, as it is strongly associated with disease progression; however, this does not translate to an effective and complete cure of the HBV infection. Among the several barriers to cure, the most worrying one is the covalently closed circular DNA (cccDNA), which allows the virus to permanently persist in hepatocytes and against which NAs have little effect [5]. Moreover, NAs rarely achieve the so-called “functional cure”, which was defined by clinical guidelines as seroclearance of hepatitis B surface antigen (HBsAg) with or without anti-HBs. Therefore, treatment is often lifelong and often leading to the selection of resistant mutants or causing side effects [4,6]. As for HDV infection, the ideal goal of treatment is the clearance of HBsAg plus a sustained HDV virological response at least 6 months after stopping the treatment, and the attainment of both the aforementioned aims is truly challenging. Pertaining to chronic HDV infection (CHD), the treatment of choice is a one year-course of PegIFNa, usually leading to a reduction of the HDV RNA viral load, but this may prove useless if not associated with a clearance of the HBsAg [7]. When compared to other viral chronic hepatitis, there are certainly fewer data on the PegIFNa efficacy for chronic hepatitis D. To date, the largest available trial includes a total of 38 participants. Treatment success was achieved in only eight patients (21%) after 24 weeks of follow-up (all patients were maintained on PegINFa for 48 weeks) [8]. A higher virological response rate (43%) after a 12-month-follow-up was instead found in a subsequent trial. Nevertheless, it was carried out in a restricted group of 14 patients [9]. The European Association for the Study of the Liver (EASL) guidelines suggest tenofovir or entecavir treatment for those patients not eligible for interferon-based therapy with detectable HBV DNA levels in order to block residual HBV replication, mainly in patients with decompensated liver disease [4]. Unsurprisingly, rather than an ineffective drug with a well-known toxicity, we support the search for new molecules.

Although there are multiple comprehensive literature reviews on chronic hepatitis B and D treatments, there are still several critical issues, which frequently remain uncovered.

As CHB is associated with aging population, individuals often have co-morbid health concerns. Although current and investigational therapies do not carry high risks of toxicities, attention should be paid to subsets of the population called special populations, such as HIV coinfected patients, children, pregnant women, immunosuppressed patients, and patients undergoing chemotherapy and dialysis. Moreover, the futility of long-term NA therapy has become a very interesting topic, and the approach of finite NA treatment is not completely uniform.

The purpose of our study is not only to overview the different therapeutic options for chronic hepatitis B and D but to focus on those critical issues especially.

## 2. Overview of the Drug Pipeline

To succeed in the cure of the chronic infection, the prevailing theory at the moment is that the combination of two different strategies is required [3,10]. On the one hand, the recent progress in understanding the structure and life cycle of the virus allowed the development of novel antivirals directly targeting multiple steps in the virus replication, preventing the synthesis of new cccDNA. On the other hand, immunomodulators are also needed to subvert the state of tolerance found in the chronic phase of the disease and consequently promote the death of infected hepatocytes and neutralization of circulating virions [5]. According to the latest update, more than 50 compounds are currently being tested for CHB, and the majority of the studies are in a preclinical phase [11].

Adding to an existing class of drugs, new nucleotides analogs in development include besifovir, metacavir and two prodrugs of tenofovir (tenofovir exalidex, tenofovir disoproxil orotate). However, it is now widely recognized that an efficacious therapy should target more than one step of the virus replication cycle. For this reason, other drugs currently in development include attachment/entry inhibitors, such as bulevirtide.

Myrcludex-B (Myr), also known as bulevirtide, acts upon the sodium taurocholate cotransporting polypeptide (NTCP), a receptor of both HBV and HDV. Therefore, this new drug might block HBV and HDV entry, and it was approved in the European Union in July 2020 as the first effective drug for the treatment of chronic HDV in patients with compensated liver disease [11,12]. In a phase 2a trial, patients were treated with Myr for 72 weeks, and a follow-up was planned 6 months after the end of treatment. The estimation of efficacy parameters was planned to be performed after 24 and 48 weeks of therapy and after the end of follow-up. The results, though, were published as interim findings at week 24 and showed that all patients with measurable HDV RNA experienced a decline of HDV RNA under Myr monotherapy, while, remarkably, the combination of Myr with PegIFNα-2a profoundly enhanced this antiviral effect, achieving a decline >1 log in HDV RNA in all the subjects. Finally, ALT levels significantly declined in six of the eight patients of the Myr cohort [13]. Regarding the reduction of >0.5 log HBsAg, which we already described as an alternative therapeutic target, none of the patients achieved this endpoint. Similarly, Wedemeyer et al. showed a HDV RNA declined by 2 log and a normalization of ALT levels in patients treated with Myr and tenofovir, but regrettably, HDV RNA replication relapsed after the end of treatment in most of the patients and HBsAg remained unaffected [14]. Moreover, recent studies showed that the effect on HBsAg seemed to be more pronounced in the HDV patients receiving lower doses of Myr in combination with IFNa, rather than higher. The reason for this observation is not currently known.

Lonafarnib (LNF) is a farnesyl transferase inhibitor, which blocks assembly and secretion of virions in the cell (IC50: 36 nM) through the hepatitis delta antigen prenylation. LNF has been more extensively studied because of its potential activity in cancer patients and its proven efficacy in Hutchinson–Gilford progeria syndrome. In a phase 2a study, 14 patients were randomly assigned to receive LNF 100 mg or 200 mg twice daily for 28 days with greater decline in HDV RNA [15]. In a subsequent study, Yurdaydin et al. explored different potential LNF regimens: different doses of LNF, LNF plus ritonavir (RTV), LNF plus PEG-INF. A better antiviral response was achieved with the addition of RTV supporting the key role of the cytochrome P450 3A4 inhibitor and the need of exploration of boosting combinations [7].

Recently, nucleic acid polymers (NAPs), such REP 2139, have also been widely studied, showing promising results, as after a follow-up of 1 year, 7 and 5 of the 12 evaluated patients were HDV RNA and HBsAg negative, respectively. Asymptomatic and transient elevation of liver enzymes have been also reported [16]. Similarly, PEG-IFN-lambda was associated with improved or similar rates of virologic response with fewer adverse events than IFNa. The primary end point was once again a reduction of >2 log or negative HDV RNA at the end of 48-week-treatment and following a 24-week observation period [17]. We underline here that therapeutic targets remain generally similar in the older and newer studies assessing the efficacy of HBV or HDV treatment. In any case, depending on the treatment aim (HBV DNA or HBsAg decline, HDV-RNA long-term suppression, ALT normalization, etc.) and the degree of hepatic impairment, these novel regimens might potentially be successful, and with additional strategies, such as drug combinations, they might work even better.

Other molecules working with different mechanisms include:

- siRNAs (small interfering RNA that interfere with viral mRNA to prevent synthesis of viral antigens): GalNAc-siRNA, VIR-2218, DCR-HBVS, JNJ-3989, ARB-1467. Mostly now in phase 1 or 2 studies.
- Antisense nucleotides: GSK3389404, RO7062931, GSK3228836. In phase 2 studies.
- RNase H targeting (prevents degradation of pre-genomic RNA and synthesis of DNA): a-hydrocytropolones, N-hydroxyisoquinolinediones, N-hydroxylpyridinediones. These are the chemical class of molecules now under investigation, no single molecule has been developed yet.
- Capsid inhibitors (interfere with the formation of the HBC core protein): GLS4, JNJ 56136379, JNJ 56136379 (alone or in combination with JNJ 73763989), ABI-H0731, ABI-H2158, QL-007, RO7049389, EDP-514, AB-423, and JNJ-6379. Mainly in phase II, some in phase I or in vitro studies, alone or in combination with nucleotide inhibitors. A study of a capsid inhibitor in combination with Toll-like receptor 7 agonist is also in program (RO7049389 + RO7020531).
- HBsAg release inhibitors (prevent the assembly and secretion of HBV subviral particles: REP 2139 (also in combination with REP 2165), REP-2055, REP 301, REP 301-LTF, REP 401, REP 102. Some of these have been studied in combination with Peg-IFN and TDF. Now in phase II.
- cccDNA formation inhibitors: ccc_R08. Now in animal studies [5].

In the immunotherapy side of HBV treatment, mechanisms and molecules under study include:

- Toll-like receptor agonists (activation of innate immune system with production of IFN): GS-9620, GS9688, TQ-A3334, RO6864017. As explained above, there is also RO7020531 in combination with a capsid inhibitor (RO7049389 + RO7020531). They are mostly in phase II.
- Retinoic acid-inducible gene-1 agonist (lead to production of IFN and other cytokines that activate antiviral immunity): Inarigivir, SB-9200.
- Agonists of IFN genes stimulators (IFN production). Now in animal studies.
- Checkpoint inhibitors (restore T-cell functionality): CTLA-4, CD244/2B4, Tim-3, LAG-3, HLX10, cemiplimab, nivolumab in combination with a therapeutic vaccine.
- Therapeutic vaccines: ABX-203, INO-1800 (with or without INO-9112), HB-110 (with adefovir), GS-4774, TG-1050, JNJ-64300535, FP-02.2, DV-601, HBV0003, T101, GC1102. Mostly in phase I.
- Apoptosis inducers: APG-1387
- Ciclophilin inhibitor: CRV-31
- Transfer of genetically engineered T cells or CAR (chimeric antigen receptor) T cells [5].

## 3. Pharmacology and Safety of Current and Investigational Therapies of Hepatitis B and D

### 3.1. Myrcludex B (Myr)

Myr targets the hepatocytes exclusively, and this might allow subcutaneous administration of low drug doses [18]. Phase III clinical trials have established a subcutaneous injection of 10 mg as the optimal dose to reach more than 80% saturation of the NTCP receptor for at least 15 h [19]. The raised concern that NTCP blockage might cause an elevated plasma bile acid levels-related adverse reaction [20] is now insubstantial because, while the inhibition of HBV/HDV infection is reached with an inhibitory concentration (IC) 50 of 80 pM [21], the increase in bile acid transportation is impaired with an IC 50 of 47 nM, therefore significantly higher. Hence, Myr effectively inhibits HBV/HDV infection at concentrations where the NTCP-mediated transport of substrates is not yet affected. However, whether NTCP inhibition can also affect drug exposure is unknown. Conversely, plasma bile acid levels might work as the drug’s marker. A study by Blank et al. recently investigated the pharmacokinetic data of Myr, and its effects on TDF 300 mg in 12 healthy volunteers after administration of a 10 mg SC dose. The authors noted that the steady-state AUC and the Cmax were significantly higher compared with those following the first dose, thus indicating an accumulation [19]. A further major consideration for clinical practice concerns the drug’s excretion, and renal excretion resulted as a negligible route of elimination of Myr [22].

A critical issue is definitely the combination of antiviral therapies for hepatitis B infection. The reason behind it is precisely to achieve the HBsAg loss, acting at different stages of the disease, and simultaneously decrease HBV attachment and entry, ccc DNA formation, nucleocapsid and core assembly. For example, although Myr blocks viral entry, HDV and HBV can still propagate undisturbed through cell division, which is, conversely, efficiently restricted by IFN. IFN-α inhibits HBV transcription and replication in cell culture and in humanized mice by targeting the epigenetic regulation of the nuclear cccDNA minichromosome [23,24]. In the published first results of a phase 2 trial, a benefit of the antiviral combination of PegIFNα-2a and myrcludex was definitely observed [13]. However, follow-up showed a viral rebound after treatment cessation. Myr may thus be combined with current HBV drugs to improve HBV or HBV-HDV infected patient outcome; however, despite a decrease in HDV-RNA in a dose-dependent manner, only 10% of patients treated with Myr showed a definite virological response (defined as a 2 log10 reduction in HDV-RNA). The optimal duration of treatment to clear HDV RNA permanently is still unknown, since studies of 2 to 3-year duration are being planned, while the suggestion of potential benefit of a higher dose of Myr has been investigated by Loglio et al. [25].

### 3.2. Lonafarnib

Lonafarnib (LNF) is a farnesyl transferase inhibitor, which blocks assembly and secretion of virions in the cell (IC50: 36 nM) through the hepatitis delta antigen prenylation [15,26]. LNF has dose and time-dependent pharmacokinetics with an insignificant renal excretion [27]. Moreover, this drug notoriously has some adverse events, mostly in the multiple-dose rather than once-daily administration, mainly reported as minor gastrointestinal disorders, which significantly decreased with food intake [28]. Although the recommended dose is 200 mg bid [29], a recent PK and PD study showed that a high LNF dose of 610 mg bid would achieve 99% efficacy. However, such a high dose might cause several adverse effects [30]. Therefore, the authors provided an already explored suggestion regarding the use of a ritonavir booster to potentially optimize both the LNF tolerability and its antiviral effect [31]. The true ramifications of this option will need to be extensively investigated. Finally, the work of Lempp and colleagues indicates that, besides the suppression of viral secretion, LFN led to an intracellular accumulation of a hepatitis delta antigen [26].

### 3.3. JNJ-56136379

JNJ-5613379 (JNJ-6379) is an oral drug, which has at least two mechanisms of action on HBV infection. First, it interferes with the HBV capsid assembly, and second, it prevents cccDNA formation during de novo infection. Recently, Vandenbossche et al. demonstrated a dose-proportional increase in plasma concentration and AUC of the drug administered to healthy subjects [32]. However, this is true for dosages up to 300 mg, while with a double dose of 600 mg, the clearance decreased, determining a less than dose-proportional increase in the drug. Moreover, the drug showed a very long half-life of 120–140 h. Significantly, the drug clearance also decreased with lower weight [32]. No clear information regarding the metabolism of this drug is currently available. However, since a renal excretion of 18% has been recently reported, a certain share of hepatic metabolism probably exists. How this might potentially result in a drug–drug interaction (DDI) is still unclear [33]. Importantly, no severe adverse reactions were reported in the first in vivo single and multiple dose trial in healthy volunteers, and there was no dose limiting toxicity [34]. Only one patient experienced an elevation of ALT and AST during the treatment, but it was not possible to link it with any certainty to therapy [35]. A phase 2, randomized, open label study is currently ongoing to evaluate efficacy, pharmacokinetics, safety and tolerability of response-guided treatment with this drug combined with NA and Pegylated Interferon Alpha-2 [36].

### 3.4. ABI-H0731

ABI-H0731 is an orally administered, HBV core protein inhibitor, which also blocks several other steps in the HBV life cycle, including the HBV DNA synthesis and cccDNA formation. For this very reason, core inhibitors might have a more profound inhibitory effect on overall HBV replication than nucleoside analogs alone [37,38]. In a recent randomized, placebo-controlled, first-in-human study by Man-Fung Yuen et al., ABI-H0731 pharmacokinetics were assessed in healthy volunteers and HBV chronic patients. Overall, the authors’ aim was to identify a safe and effective dosing schedule for phase 2 clinical studies [39]. Interestingly, this study showed that ABI-H0731 has dose-proportional pharmacokinetics, since steady-state Cmax, Cmin, and AUC increased when a higher dose of the drug was administered. The drug was rapidly absorbed, with mean time to maximum plasma concentration (Tmax) values of 2 × 50–4 × 17 h, and inter-individuals’ variability in pharmacokinetic parameters was low. Furthermore, a moderate-fat meal intake has a significant impact on absorption, causing an approximately 45% increase in AUC. These findings are supportive of once-daily dosing of this drug. Nevertheless, it is interesting to note that chronic HBV patients experienced a higher exposure to the same dosages of ABI-H0731 than healthy individuals, suggesting a currently unexplored hepatic metabolism of the drug and hanging question marks over cirrhotic patients on the one hand, and potential drug–drug interactions on the other. Regarding the efficacy of ABI-H0731 in chronic HBV, when administered as monotherapy for 28 days (and 28 days of follow-up), the drug exhibited a dose-related antiviral activity, with mean maximal HBV DNA decline from baseline of 1 × 7 log10 IU/mL at 100 mg to 2 × 8 log10 IU/mL at 300 mg after 28 days, for both HBeAg positive and negative participants. To further confirm that a combination therapy is preferable, a more profound HBV DNA decline of treatment was seen when patients were treated with both an NA and ABI-H0731, compared with the placebo [40]. Therefore, the combination might not only maximize the antiviral potency but also avoid treatment-emergent resistance. Regarding the safety data, while a macular/maculopapular rash should be considered during the treatment, since some moderate cases occurred, the treatment was well tolerated overall [39].

### 3.5. REP-2139

REP-2139 is a nucleic acid polymer (NAP), which acts as a secretion inhibitor. The currently available studies investigated its role as monotherapy and in combination with NA or IFNa for 24–48 weeks, either IV or SC [16,41]. The suggested role of this compound is the removal of HBsAg from the blood, unmasking the anti-HBs response, and finally allowing the HBV clearance. Moreover, leading to a favorable immunological activation in the absence of HBsAg, this drug would potentially enhance the effect of IFNa and TDF [16,41]. Even considering all chance-related uncertainties, and due to the lack of pharmacokinetic data on REP-2139, its relative resemblance to other compounds under current use for different conditions, such as mipomersen for homozygous familial hypercholesterolemia, might lead us to consider similarities in terms of absorption, distribution, metabolism and elimination. Hence, it showed a dose-dependent maximum plasma concentration at the end of a 2-h IV infusion or SC administration, while the time of peak concentrations (t max) were typically observed 3–4 h after SC dosing and the half-time was quite long, with post-distribution-phase plasma concentrations well predicting tissue concentrations and pharmacological activity [16]. Regarding REP-2139, some data on safety are currently available. Administration-related side effects, including fever and chills, were commonly experienced but generally did not require specific therapy. As in all oligonucleotides, an improved tolerability was then attributed to the neutralization of the chelation of calcium or magnesium. Importantly, significant elevation flares of ALT and AST (>10X ULN) were frequently observed during REP-2139-Ca monotherapy in HBV/HDV patients, treated either with monotherapy or combination therapy [42]. This phenomenon, though, was self-limited and so did not require any dose adjustment and/or interruption of treatment (Table 1).

## 4. Evaluating the Response to the Hepatitis D Treatment

The only recognized available and effective drug against HDV is interferon alfa (IFNa), and it is recommended for patients with detectable HDV RNA and active liver disease (elevated serum aminotransferase and/or chronic hepatitis on liver biopsy) [43]. The treatment of chronic hepatitis D remains unsatisfactory and the eradication of HDV and HBV and prevention of the long-term sequelae of chronic hepatitis, such as cirrhosis, liver decompensation, and HCC are still not commonly achieved. Since the primary endpoint of treatment is suppression of HDV RNA, the standard of therapeutic success was defined as negative HDV RNA at 6 months (24 weeks) or more after treatment, known as a sustained virological response (SVR) [9,44]. Detectable HDV RNA at 6 months of treatment might be a predictor for a failed virological response [45]. Although achieving a negative HDV viremia is still considered a hallmark of treatment efficacy, several studies have shown that the only robust endpoint might differently be the clearance of the HBsAg [46,47]. It finally seems that the ideal goal of HDV treatment should be both the clearance of HBsAg and the sustained HDV virological response at least 6 months after stopping the treatment, and the attainment of both the aforementioned aims is truly challenging [48]. Moreover, together with a negative HDV viremia, a successful treatment is also associated with amelioration of necroinflammatory activity, defined as a sustained biochemical response (normalization of ALT and/or AST levels at six months or more after treatment) or histological response (improvement of inflammatory activity confirmed by liver biopsy). These goals are commonly considered as secondary outcomes measures in the available trials [45]. Taking these into account, Yurdaydin et al. recently proposed the evaluation of HDV treatment success based on the improvement of liver function rather than the virologic response. Relying on this unorthodox method, a decline of two or more logs of HDV RNA even without achieving a negative HDV RNA test might be sufficient, if ALT are normalized [7].

Therapeutic targets remain generally similar in the older and newer studies assessing the efficacy of HDV treatment. In any case, depending on the treatment aim (HDV-RNA long-term suppression, ALT normalization, etc.) and the degree of hepatic impairment, these novel regimens might potentially be successful, even more if additional strategies, as the combination of drugs, are implemented. Our expert opinion is that the primary goal would be, first of all, the functional cure of HBV.

## 5. Finite Nucleos(t)ide Analog Therapy

Long-term therapy with NA is effective in achieving viral suppression; however, this is not indicative of HBV eradication [49]. As highlighted by Papatheodoridi et al., several reasons have driven the emergent proposal to stop long-term NA therapy: the futility of continuing a therapy that does not offer any further benefit; the unknown safety of a lifetime NA therapy; the cumulative cost; the undoubted risk to occur through a decline of treatment adherence [50]. Despite the lack of a well-defined endpoint for HBV treatments, international guidelines unanimously consider HBsAg loss as the most important one [6]. However, Dusheiko et al. reported that HBsAg loss rates were <1% per year during NA treatment [6,51]. With this in mind, over the past 5–10 years, the finite NA treatment became a very interesting topic [6], and since 2016, the international guidelines have begun accepting finite NA therapy as an option in a specific subset of patients.

Although the approach of finite NA treatment is not completely uniform, there is a consistent agreement among different guidelines: finite NA therapy was suggested in not-cirrhotic patients with undetectable levels of HBV DNA(on three separate occasions, 6 months apart) after 12–18 months from HBeAb seroconversion (consolidation therapy) [52]. The Asian guidelines, in contrast to EASL and AASLD, consider finite NA treatment also in patients with cirrhosis [4,53,54,55]. After discontinuation, virological relapse is quite common; however, not all patients necessarily have a biochemical relapse, which means that not all patients require retreatment [53]. Although several studies have focused on identifying factors that might predict relapse after treatment discontinuation, at present, there is no reliable marker able to predict such a response [53]. Liu Y et al. performed a meta-analysis to evaluate those factors and data showed that older age, high levels of quantitative HBsAg (>1000 IU/mL) at baseline and at the end of treatment, and shorter duration of consolidation therapy result as factors predictive of relapse after NA discontinuation in HBeAg-negative CHB patients [56]. Certain viral markers have gained interest, such as the hepatitis B core-related antigen (HBcrAg), which seemed to serve as a useful marker on patients who are planning finite NA therapy. In particular, a decrease in HBcrAg levels was reported during NA therapy; Matsumoto et al. reported an experience from CHB patients treated with lamivudine where HbcrAg levels > 4.9 log U/mL at the time of NA discontinuation were correlated to clinical relapse. In contrast, HbcrAg levels < 3.4 log U/mL were the only independent predictive factor without relapse after NA cessation [57].

Moreover, immunological studies have highlighted the role of the host immune response as a pathobiological basis to facilitate HBsAg decline towards HBsAg loss [58]. Considering that, investigators have progressed to exploring immune biomarkers [53], such as soluble isoform of growth stimulation expressed gene 2 (ST2), which belongs to the Toll-like/interleukin-1 receptor superfamily. Xie et al. showed that ST2 was correlated with HBsAg, HBV DNA, ALT and anti-HBc levels. Although baseline levels of ST2 were not associated to clinical relapse, after 12 weeks after NA cessation, the level of ST2 was able to predict the clinical relapse [59].

### 5.1. When and Whom to Stop Long-Term NA Therapy?

After reviewing the current literature, we suggest that NAs should be discontinued:

- After HBsAg loss; [4]
- In patients with HBV DNA undetectability after 12–18 months from HBeAb seroconversion (consolidation therapy): [4];
- In HBsAg-positive patients without liver cirrhosis who achieved stable HBV DNA undetectability on three separate occasions, 6 months apart after treatment for 2–3 years [4,6].

Conversely, NAs discontinuation should be avoided in:

- Cirrhotic patients;
- Patients who are not motivated to adhere to close monitoring;
- Patients with HIV or HDV coinfection [6].

As already pointed above, predictors of post-NA relapse are lacking; however, Papatheodorid et al. showed an overview of biomarkers able to identify non-cirrhotic CHB patients who can safely discontinue NAs before HBsAg loss; HBsAg serum levels at NA discontinuation seem to be able to predict the clinical relapse, as already emerged from the meta-analysis of Liu Y et al. [53,56] (Table 2).

### 5.2. Management of Patients after NA Cessation

Concerning the management of patients after NAs cessation, liver function tests (serum ALT/AST, bilirubin and prothrombin time) should be monitored at week 6, week 12, week 18, week 24, and 3 monthly thereafter for the first 2 years. Weekly or biweekly tests are recommended in the case of elevation of ALT or AST > 5X ULN.

HBV DNA and HBsAg should be monitored every 3 months in the first year or in case of virologic relapse or clinical relapse and every 6–12 months afterward [6,58].

## 6. Special Populations

Current guidelines for special populations in HBV-infected patients include HIV-HBV coinfected patients, children, pregnant women, immunosuppressed patients and patients undergoing chemotherapy and dialysis. In most cases, practice points are well defined: HIV patients should follow a TAF/TAF based regimen; safety and efficacy profiles for interferon, TDF and entecavir in children are similar to adults, allowing for easy treatment when warranted; HBsAg-positive patients undergoing immunosuppressive therapy should undergo prophylaxis according to their reactivation risk; renal failure/dialysis patients should be administered entecavir or TAF as treatment/prophylaxis. Studies regarding new drugs, are, however, still missing. A search on clinicaltrial.gov reveals that active studies regarding bulevirtide either exclude special populations or do not plan sub-analysis for them. Similarly, Pubmed searches for “bulevirtide + HIV”, “bulevirtide + pregnancy”, “bulevirtide + children” and similar or related keywords yields no results. Lonafarnib has been more extensively studied (though evidence is still scarce) because of its potential activity in cancer patients and its proven efficacy in Hutchinson–Gilford progeria syndrome. Studies on solid tumors and hematological patients include the following pathologies: myelodysplastic syndrome, chronic myelomonocytic and myelogenous leukemia, lymphoma, breast, central nervous system, gastrointestinal, genitourinary, head and neck, lung and liver cancer and soft tissue sarcomas. Most of these studies are still ongoing with no published results; however, some phase II studies that used standard lonafarnib dosage (100 to 200 mg twice daily) reported mainly grade 1 and 2 side effects, with no excess of hematological side effects when compared to standard therapy [60,61,62,63,64,65]. A case report in three chronic myelomonocytic leukemia patients describes hyperleukocytosis associated with respiratory distress that has not been observed in other patients [27,66,67,68,69,70,71]. Thus, we can conclude that in terms of side effects, cancer patients and patients undergoing chemotherapy require no special attention. A few studies also examined potential drug–drug interactions, finding no interactions with gemcitabine, imatinib, paclitaxel. Concerning children, a phase I study on pediatric cancer patients determined an optimal body surface area-dependent dose (yielding good serum levels with grade 1 or 2 side effects) similar to adults, and higher doses resulted in the same side effects. It also noted that myelosuppression occurred only with higher doses, as it happened in adults [72]. This might also make this drug ideal for immunosuppressed patients. Studies on Hutchinson–Gilford progeria syndrome were obviously directed at children, and showed pharmacological properties and side effects profiles similar to the ones observed in adults [73,74]. Obviously, children with Hutchinson–Gilford progeria syndrome represent a nearly unique category given the rarity of the disease, and this, plus the minuscule number of participants involved in these studies, do not guarantee reliability of the findings on pharmacodynamics-kinetics and uncommon side effects. Other drugs in the pipeline for HBV treatment are still too new in their development phases to have data about special populations.

## 7. Conclusions

In this paper, we provided an overview of the different therapeutic options for chronic hepatitis B and D. As discussed above, despite the accumulating knowledge, although HBV and HDV infections represent a global health problem, unmet clinical needs still remain. Firstly, the chance of a cure with the currently available antiviral drugs is very low. Secondly, as infections are associated with an aging population, individuals often have co-morbid health concerns. Although current and investigational therapies do not carry high risks of toxicities, attention should be paid in a subset of the population called a special population, such as HIV coinfected patients, children, pregnant women, immunosuppressed patients, and patients undergoing chemotherapy and dialysis.

In view of this, a basic understanding of actions and safety of current and investigational therapies should be useful to guide clinicians toward the correct therapeutic choice. Further studies will focus on the development of combination strategies targeting different signaling pathways towards a functional cure, most probably with a combination of multiple drugs.

## Figures and Tables

**Table 1:** Summary of pharmacology and safety of current and investigational therapies of hepatitis B and D.

**Myrcludex B (Myr)**	Injections by SC route Recommended dose is 10 mg qd Insignificant renal excretion
**Lonafarnib (LNF)**	Oral drug Recommended dose is 200 mg bid Insignificant renal excretion Gastrointestinal disorders, which significantly decrease with food intake
**JNJ-6379**	Oral drug Long half-life of 120–140 Renal excretion and hepatic metabolism may exist (potential DDI)
**ABI-H0731**	Oral drug A moderate-fat meal intake has a significant impact on absorption, supportive of once-daily dosing Hepatic metabolism (potential DDI)
**REP-2139**	Injections IV or SC Side effects reported, including fever and chills

**Table 2:** Patients that should stop long-term NA therapy and when.

NAs Should Be Discontinued	NAs Discontinuation Should Be Avoided
HBsAg loss	Cirrhotic patients
After 12–18 months from HBeAb seroconversion with HBV DNA undetectability	Patients who are not motivated to adhere to close monitoring
HBV DNA undetectability on three separate occasions, 6 months apart after treatment for 2–3 years	Patients with HIV or HDV coinfection
